# Interphase Effect on the Macro Nonlinear Mechanical Behavior of Cement-Based Solidified Sand Mixture

**DOI:** 10.3390/ma15051972

**Published:** 2022-03-07

**Authors:** Fengxue Wang, Yan-Gao Hu, Li Liu, Yongfeng Deng, Shuitao Gu

**Affiliations:** 1 Department of Chongzuo Housing and Urban-Rural Development Bureau, No. 21 Detian Road, Chongzuo 532200, China; fxwang@cqu.edu.cn; 2 Department of Civil Engineering, Chongqing University, No. 83 Shapingba North St., Chongqing 400045, China; yangaohu@cqu.edu.cn; 3 Department of Key Laboratory of New Technology for Construction of Cities in Mountain Area, Ministry of Education, No. 83 Shapingba North St., Chongqing 400045, China; 4 Department of Geotechnical Engineering, School of Transportation, Southeast University, No. 2 Jiangning Rd., Nanjing 211189, China; liliuseu@seu.edu.cn

**Keywords:** cement-based solidified sand mixture, interphase effect, numerical simulation, macro elastic modulus, unconfined compressive strength, damage

## Abstract

This paper investigates the interphase effect on the macro nonlinear mechanical behavior of cement-based solidified sand mixture (CBSSM) using a finite element numerical simulation method. CBSSM is a multiphase composite whose main components are soil, cement, sand and water, often found in soft soil foundation reinforcement. The emergence of this composite material can reduce the cost of soft soil foundation reinforcement and weaken silt pollution. Simplifying the CBSSM into a three-phase structure can efficiently excavate the interphase effects, that is, the sand phase with higher strength, the cement-based solidified soil phase (CBSS) with moderate strength, and the interphase with weaker strength. The interphase between aggregate and CBSS in the mixture exhibits the weak properties due to high porosity but gets little attention. In order to clarify the mechanical relationship between interphase and CBSSM, a bilinear Cohesive Model (CM) was selected for the interphase, which can phenomenologically model damage behaviors such as damage nucleation, initiation and propagation. Firstly, carry out the unconfined compression experiments on the CBSSM with different artificial gradations and then gain the nonlinear stress–strain curves. Secondly, take the Monte Carlo method to establish the numerical models of CBSSM with different gradations, which can generate geometric models containing randomly distributed and non-overlapping sand aggregates in Python by code. Then, import the CBSSM geometric models into the finite element platform Abaqus and implement the same boundary conditions as the test. Fit experimental nonlinear stress–strain curves and verify the reliability of numerical models. Finally, analyze the interphase damage effect on the macroscopic mechanical properties of CBSSM by the most reliable numerical model. The results show that there is an obviously interphase effect on CBSSM mechanical behavior, and the interphase with greater strength and stiffness ensures the macro load capacity and service life of the CBSSM; a growth in the interphase number can also adversely affect the durability of CBSSM, which provides a favorable reference for the engineering practice.

## 1. Introduction

CBSS appears extensively in soft soil foundation reinforcement projects for roads, railways and buildings [1]. However, damage and cracking of CBSS foundations can easily destroy the performance, durability and service life of roads and building structures. When the CBSS foundation is subjected to a mechanical or environmental load for a long time, cracks are prone to occur. These cracks usually manifest as transverse and longitudinal cracks in the foundation surface, which will lead to water penetration, the deterioration of the internal structure of the foundation, and thus the service life of the foundation being seriously impaired. Even if the foundation is covered and repaired, the hidden cracks will eventually cause the failure of the covering.

To improve the mechanical properties of CBSS, a method of adding sand has been focused on [2]. The CBSSM is a kind of multiphase material composed of soil, cement, sand and water, prepared by uniformly stirring soil, sand and cement mixed in a certain proportion, and adding water corresponding to the moisture content. After a fixed age of curing, the composite can be regarded as sand, cement hydration, clay, interphase and pores. According to the distinction of mechanical properties, we can describe it as having three phases, namely the sand phase with higher strength, the CBSS phase with moderate strength, and the interphase with weaker strength. Among them, cement hydration products, clay and pores make up the CBSS phase. The interphase between the CBSS and the sand is characterized by high porosity, low hardness and rich hydration products such as CH (calcium hydroxide) [3], which is of great significance for the mechanical properties and fracture process of composite materials [4]. The initial microcracks and the stress concentration result in a lower bond strength of the interphase in the three phases [5,6]. Rao and Prasad [6] pointed out that the cracks of the cementitious composites should firstly occur in the weakest interphase, and then propagate to the cement solidified phase and aggregate phases. Jin et al. [7] also indicated the interphase strength is a factor that cannot be ignored. As such, it is crucial to acknowledge the role of the interphase, which will vividly clarify the damage path and failure mechanism of composite materials and assist in proposing corresponding improvement schemes. However, for the CBSSM, the effect of the interphase damage process on macroscopic mechanical behavior has not been sufficiently concerned and needs to be further explored.

To study the interphase effects on the composite macro mechanical behavior, the spring layer model is widely recognized in the linear elastic framework for cementitious composites [8,9,10,11]. He et al. [12] introduced the concurrent algorithm-based discrete element system to simulate the densely packed structure of an arbitrary-shaped aggregate, the increase of the macro elastic modulus was addressed by incremental interphase’s modulus. Zhang et al. [13] believes that it is worth paying attention to the interphase effect on direct tensile strength. In addition, the interphase effect on macro mechanical properties is also controlled by the aggregate volume fraction [14,15,16]. However, a series of studies are based on the spring layer model, which is limited to the interphase linear elastic properties and cannot involve the interphase debonding along with the interphase degradation, especially for this special material CBSSM. Linear Elastic Fracture Mechanics (LEFM) methods are traditional for fracture characterization of cementitious composites. However, the LEFM method is limited to very small brittle fractures in the fracture process zone (the inelastic zone around the crack tip). Several numerical methods based on fracture mechanics methods have been developed in the past for modeling crack nucleation and propagation in cementitious composites. These methods can be roughly divided into two different models, namely the discrete crack model and diffusion crack model. In discrete crack models, damage in the material is assumed to be confined to very small lengths, while diffusion models treat cracks as damage smeared over a representative volume. However, when the material starts to soften, the diffuse model does not work due to mesh dependency issues. In this case, the energy dissipated in the damage zone converges to zero as the mesh is refined [16]. The advantages of using cohesive elements are highlighted. Although the middle surface of the cohesive element can withstand tensile and shear strains, it cannot generate any stress. Therefore, the cohesive element can only support the traction–separation failure criterion perpendicular to the upper and lower surfaces (traction–separation laws), which provides a solution without mesh refinement, effectively reduces the number of meshes and improves computational efficiency. Interphase with CM is a well-known discrete fracture model used to simulate crack nucleation and propagation for cementitious composites. In this approach, the small and negligible width of the fracture process region and the inelastic material behavior in the process region is modeled with constitutive laws that define the relationship between traction and interfacial separation. Many other research efforts have also shown the successful application of the CM in simulating crack initiation and propagation in cementitious composites [17,18,19].

The CM provides an efficient way to model damage that occurs in the region in front of the crack tip. This method with the bi-linear constitutive laws includes displacement jumps and corresponding traction forces along the interphase, and phenomenologically addresses fracture behaviors such as crack nucleation, initiation and propagation. There are three parameters required in a bi-linear cohesive law, namely the penalty stiffness, the cohesive strength and the fracture toughness. While stiffness penalty exhibits the initial elastic behavior of the phases under the traction–separation curve before damage begins, the strength reflects the maximum stress that the phases can sustained, and the fracture toughness represents the resistance to crack propagation during damage evolution. CM allows non-linear behavior such as debonding [20]. This method effectively explains the interphase effect on the macroscopic properties of composites, including various types of loading, such as static tension and compression and dynamic tension and compression [18,19].

Numerical methods have become a very effective means for alleviating the limitations of experimental research, where the sketch model of the two-phase or three-phase framework in composite is often presented. In the two-phase framework, Nilsen and Monteiro [21] found that Hashin and Strikman’s upper and lower formulas were violated [22], while Xu et al. [23] and Liang et al. [24] proved that the sand-interphase-cementation three-phase model is more reasonable. Obviously, a focus on the interphase is necessary. In the literature, there are a lot of works devoted to exploring the interphase effect on the macro mechanical properties of the composite by the Finite Element Method [8,14,25,26]. The Finite Element Method effectively alleviates the high difficulty and high cost in routine experiments at the cement-based composite level in the laboratory. Superior computational capability has driven the development of multiscale modeling approaches to investigate the correlation between macroscopic failures and microstructural changes.

This paper differs from previous work in two ways. On the one hand, the research content focuses on soft soil foundation reinforced composite materials, that is, cement-based solidified sand mixture, which is common in soft soil areas, but the corresponding interphase research is very rare. On the other hand, the paper takes CM to describe interphase by the Finite Element Method, capture the global response and general damage pattern/crack trajectory owing to the interphase effect, and probe the effects of interphase stiffness, strength and quantity on the global response. In addition to the introduction part, Section 2 presents the CM constitutive law in detail, Section 3 depicts the process of obtaining the experimental stress–strain curve, which is picked to confirm the reliability of the finite element numerical model, Section 4 characterizes the method of establishing the numerical model, Section 5 clarifies all the parameters required for the CM to simulate the interphase, Section 6 details the global response due to changes of interphase stiffness, strength and quantity, and Section 7 summarizes the whole paper and gives an outlook.

## 2. Constitutive Model

In the present work, we chose the three-phase model of the CBSSM as the numerical model. The interphase takes the form of planar two-dimensional elements in simulation, which has a very small finite thickness. The interphase behavior of CBSSM is described by a bilinear type of CM. In a 2D model, two individual fracture modes and their mixity, i.e., mode I (open mode), mode II (shear mode) and mixed mode, are considered to describe the fracture failure under general loading conditions. Additionally, each fracture mode of a certain material has 3 fixed parameters, namely penalty stiffness, strength and fracture toughness.

The interphase goes through two stages. At the early age of loading, the interphase exhibits a linear elastic behavior, which is assumed to be isotropic and can be described by the relationship between the stress vector t and the surface strain ε as

(1) $$t=\left\{\begin{array}{c} t_{n}\\ t_{s} \end{array}\right\}=\left[\begin{array}{cc} E_{nn} & 0\\ 0 & E_{ss} \end{array}\right]\left\{\begin{array}{c} \varepsilon_{n}\\ \varepsilon_{s} \end{array}\right\}$$

where $\varepsilon_{n}$ and $\varepsilon_{s}$ are the normal and tangential surface strain components, $t_{n}$ and $t_{s}$ represent the normal and tangential stress components, and $E_{nn}$ and $E_{ss}$ mean interphase normal and tangential elastic moduli, respectively. According to Gu et al. [27], it can be inferred that $E_{nn}$, $E_{ss}$ and Poisson’s ratio (ν) satisfy the following relationship:

(2) $$E_{nn}=\frac{2E_{ss}\left(1-\nu \right)}{1-2\nu }$$

When a common criterion of stress’s quadratic interaction proposed by Hashin [28] is satisfied, the initial damage of interphase begins to develop.

(3) $${\left\{\frac{\langle t_{n}\rangle }{t_{n}^{0}}\right\}}^{2}+{\left\{\frac{t_{s}}{t_{s}^{0}}\right\}}^{2}=1$$

where $t_{n}^{0}$ represents normal peak stress; $t_{s}^{0}$ is tangential peak stress. The Macaulay brackets “‹›” is defined by ‹x› = (x − |x|)/2 for arbitrary value x. That means we accept the assumption of non-damaging process of interphase under pure compressive deformation or stress state. In other words, the normal stress component effect is only considered in the traction stress state. Additionally, under the tensile and shear mixed loading, the completely debonding stage of interphase needs to satisfy the following equation [29], which means that the interphase starts to debond when the fracture toughness satisfies the following equation.

(4) $$G_{n}^{C}+\left(G_{s}^{C}-G_{n}^{C}\right){\left(\frac{G_{s}}{G_{s}+G_{n}}\right)}^{\eta }=G_{C}$$

where $G_{n}^{C}$ refers to the normal critical strain energy release rate and $G_{s}^{C}$ is the first shear direction critical strain energy release rate; η represents an empirically derived parameter. The total critical strain energy release rate, $G_{C}$ can be calculated as ∂U/2B∂a, where U is the potential energy stored in the system, B and a represent the interphase thickness and crack length, respectively, and factor 2 refers to two crack faces. This fracture criterion has recently become more commonly used in a greater variety of engineering composites [18,19].

In the three-phase model of the cement-based solidified sand mixture, we consider the constitutive model of the cement-based matrix as a damaged elastic-plastic model with an initial damage value. Thus, under uniaxial unconfined compression test, express the uniaxial compressive stress $\sigma_{c}$ as follows [30]:

(5) $$\sigma_{c}=\left(1-d_{c}\right)E_{0}\left(\varepsilon_{c}-{\tilde{\varepsilon }}_{c}{}^{pl}\right)$$

where $E_{0}$ is the initial elastic stiffness of the cement-based matrix; ${\tilde{\varepsilon }}_{c}^{pl}$ refers to the compressive equivalent plastic strain; $\varepsilon_{c}$ is the total strain; $d_{c}$ represents the scalar initial stiffness damage variable. The corresponding yielding function proposed by Lubliner et al. [31] and modified by Lee and Fenves [30] is adopted and expressed as follows:

(6) $$a=\frac{\left(\sigma_{b0}/\sigma_{c0}\right)-1}{2\left(\sigma_{b0}/\sigma_{c0}\right)-1},\;\;0\leq a\leq 0.5$$

(7) $$F=\frac{1}{1-a}\left(\bar{q}-3a\bar{p}+\beta \left({\tilde{\varepsilon }}^{pl}\right)\langle {\tilde{\bar{\sigma }}}_{\max}\rangle -\gamma \left(-{\tilde{\bar{\sigma }}}_{\max}\right)\right)-{\bar{\sigma }}_{c}\left({\tilde{\varepsilon }}_{c}^{pl}\right)=0$$

(8) $$\beta =\frac{{\bar{\sigma }}_{c}\left({\tilde{\varepsilon }}_{c}^{pl}\right)}{{\tilde{\sigma }}_{t}\left({\tilde{\varepsilon }}_{t}^{pl}\right)}\left(1-a\right)-\left(1+a\right)$$

(9) $$\gamma =\frac{3\left(1-K_{c}\right)}{2K_{c}-1}$$

where ${\bar{\sigma }}_{\max}$ refers to the maximum principal effective stress, $\sigma_{b0}/\sigma_{c0}$ is the ratio of biaxial compressive yielding stress to the initial uniaxial compressive yielding stress; $K_{c}$ denotes the coefficient ascertain the shape of the deviatoric cross-section [32], ${\bar{\sigma }}_{c}\left({\tilde{\varepsilon }}_{t}^{pl}\right)$ and ${\bar{\sigma }}_{t}\left({\tilde{\varepsilon }}_{t}^{pl}\right)$ represent the effective cohesion stresses under compression and tension; ${\tilde{\varepsilon }}_{t}^{pl}$ is tensile equivalent plastic strain; γ appears only for triaxial compression stress states.

Assume the concrete damaged plasticity model is non-associated. Here, the flow potential G can be taken as

(10) $$G=\sqrt{{\left(\xi \sigma_{t0}\tan\psi \right)}^{2}+{\bar{q}}^{2}}-\bar{p}\tan\psi$$

where ψ is the shear dilation angle measured in the *p*-*q* plane at high confining pressure, $\sigma_{t0}$ refers to the uniaxial tensile stress at failure; ξ represents the potential flow eccentricity; $\bar{p}$ represents the effective hydrostatic pressure; $\bar{q}$ is the Mises equivalent effective stress. In addition, it should be noted that in the low confining pressure, the fracture mode is generally complex and is not a shear fracture mode. This is the reason why we determine the shear dilation angle ψ in real shear fracture mode under a relatively high confining pressure.

## 3. Test Description and Results

**Figure 1:**
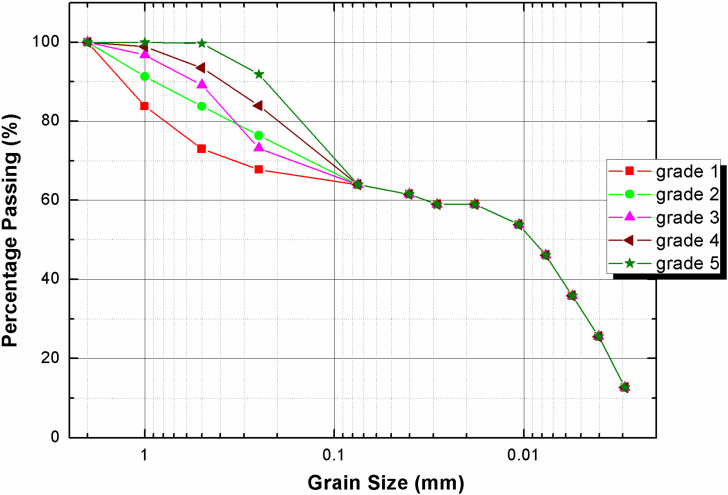
Gradation curves of sand for experiment and numerical simulation.

A series of unconfined compressive strength tests were conducted to evaluate the variations in macro-mechanical properties of the CBSSM. CBSSM is composed of soil, cement, sand and water. The soil, taken from Lianyungang City, Jiangsu Province, is marine sedimentary soft soil, the type of cement and sand are 42.5# Ordinary Portland Cement, and river sand, respectively, sands with a d50 (medium diameter, ranging from 0.16 to 0.99 mm), unevenness coefficient (equal to diameter of 60% passing to diameter of 10% passing, ranging from 0.60 to 1.34), and curvature coefficient (equal to square diameter of 30% passing to diameter of 10% and 60% passing, ranging from 1.62 to 2.96). To understand the sand gradation effect on the strength, see Figure 1 and Table 1 for gradation information. Before sample preparation, the soil samples were naturally air-dried and crushed, and passed through a 2 mm sieve. After stirring the soil sample, sand and cement evenly, add distilled water and continue to homogenize. Among them, according to the designed five-group gradation, the sand group with 48% dry soil mass, cement with 20% dry soil mass, and distilled water with 70%, 80% and 90% dry soil mass are added.

**Figure 2:**
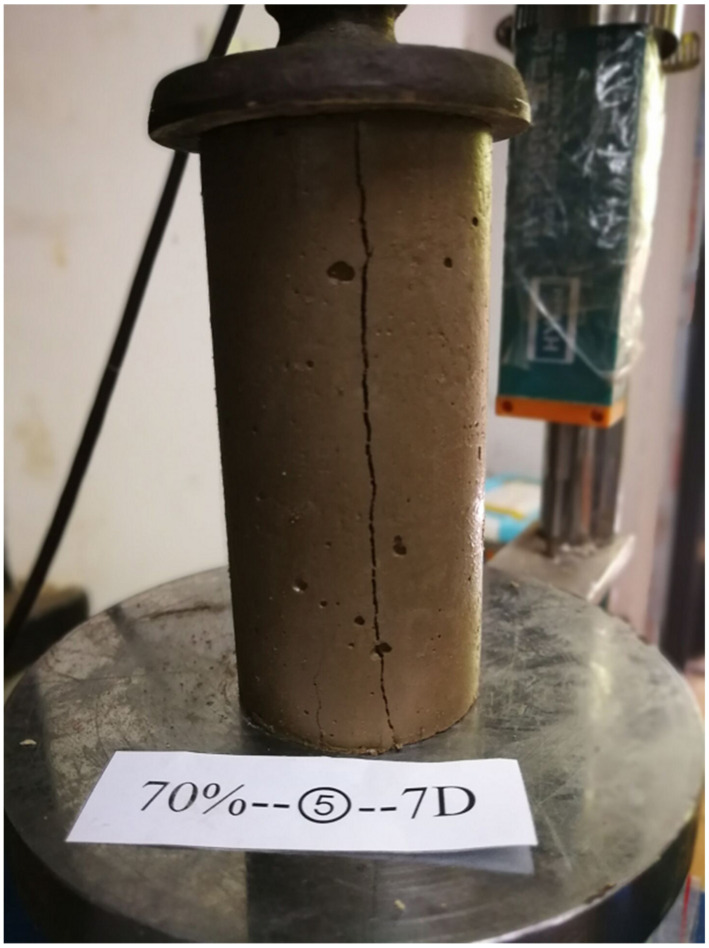
Test equipment and loading method.

After mixing, the uniform mixture was transferred to cylindrical split molds (50 mm in diameter and 100 mm in height). The mixture was artificially tapped to remove trapped air bubbles. The specimens were then sealed by plastic wraps and cured in the chamber with a temperature of 20 ± 2 °C and a humidity of 95%. Prior to testing, the top and bottom of the specimen were trimmed to ensure 100 ± 5 mm in height and to maintain a length/diameter ratio about 2:1. The mass of each sample was weighed and checked for consistency to within ±3 g of the mean mass of the mixture. At least three samples were tested for each mixture. After curing periods of 28 days, the specimens were tested for unconfined compressive strength (UCS) with a loading rate of 1.00 mm/min (ASTM D-2166) as shown in Figure 2. 

**Figure 3:**
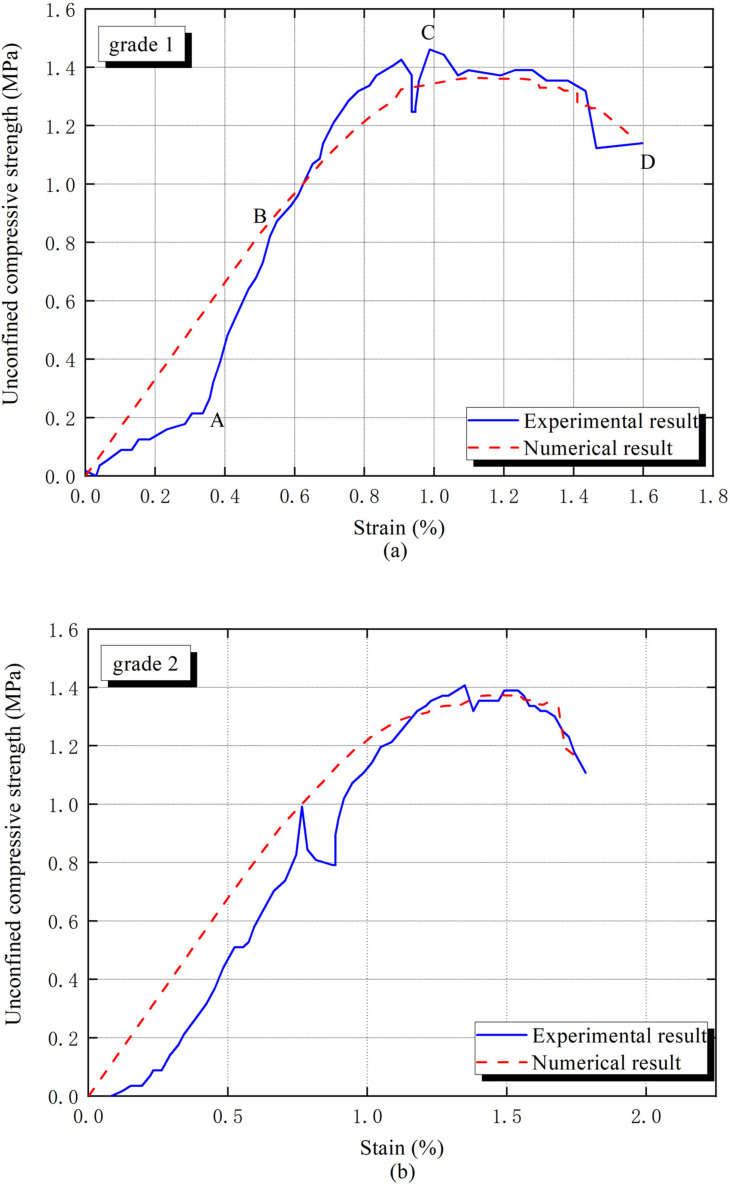

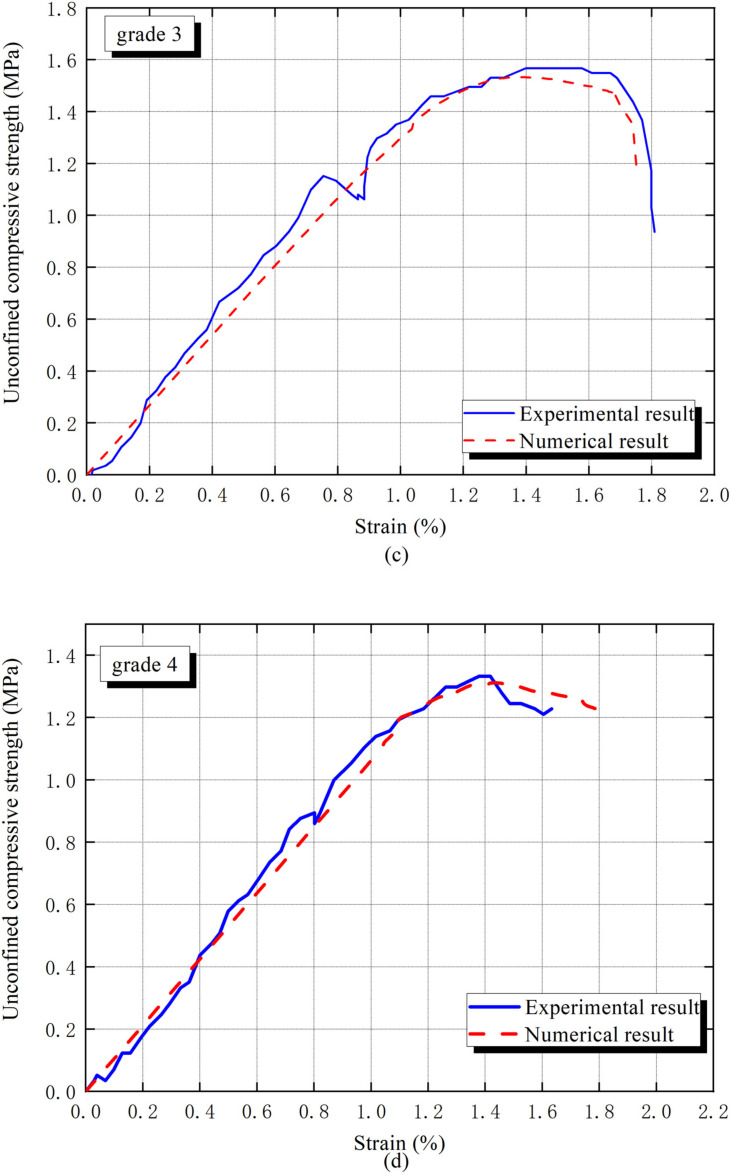
Nonlinear stress–strain curves for different aggregate gradations: Experiment and Finite element method (**a**) grade 1 (**b**) grade 2 (**c**) grade3 (**d**) grade 4.

**Figure 4:**
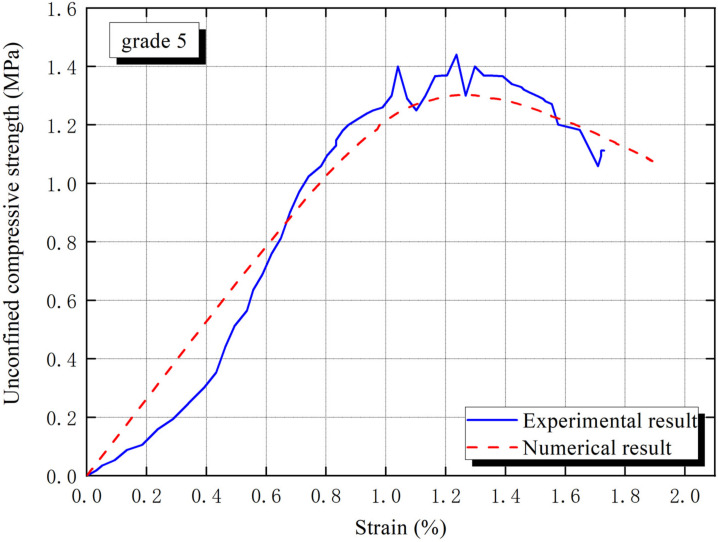
Nonlinear stress–strain curve of grade 5 for verification of interphase parameters.

Figure 3 and Figure 4 reveal the outcomes of the unconfined compressive strength test. Indicating that interphase differences such as interphase stiffness, strength and average thickness caused by gradation significantly fluctuate macro mechanical properties of cement-based solidified sand mixture.

The macro mechanical response is roughly divided into two stages in Figure 3a. In the straight line (AB), before the yielding stress B point, the solidified sand mixture material begins to soften. The reason is the weaker interphase begins to crack and a large amount of soil particles start to slip. After the stress reaches the peak (C), the interphase crack gradually penetrates and the matrix initiates cracking, meanwhile, stress decays rapidly until structural damage [33].

**Figure 5:**
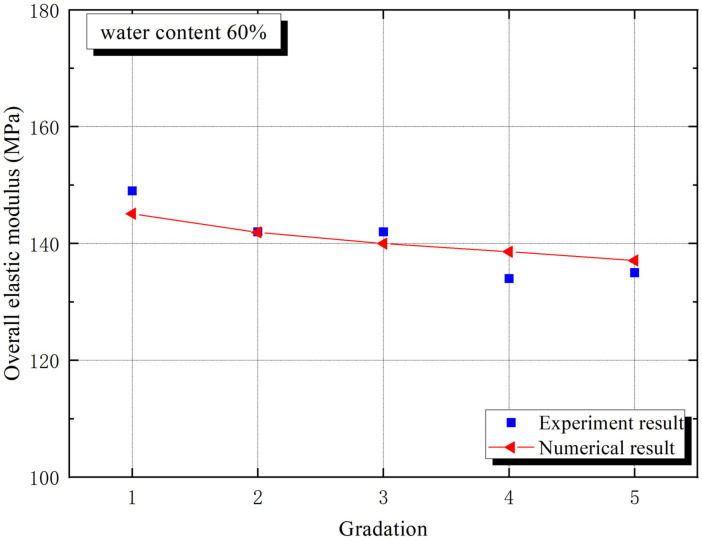
The macroscopic elastic modulus trend of CBSSM.

**Figure 6:**
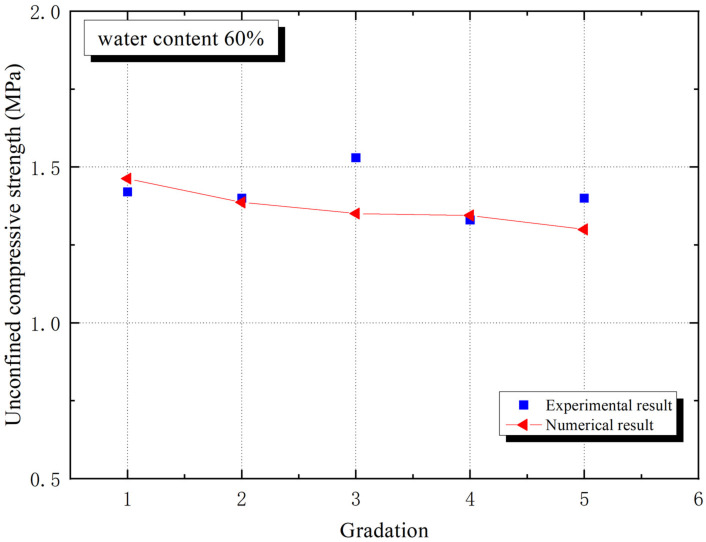
Unconfined compressive strength trend of CBSSM.

Figure 5 and Figure 6 prove the trends of macro elastic modulus and macro compressive strength with different aggregate gradations, respectively. The macro elastic modulus of grade 1 is the largest for experiment in Figure 5, while Figure 6 provides an unexpected result that the macro compressive strength of grade 3 is the largest, which indicates that sand gradation has a significant effect on macro mechanical properties. Particle size plays a role in affecting the mechanical and geometric properties of the interphase, which shows a progress.

## 4. Finite Element Simulation

**Figure 7:**
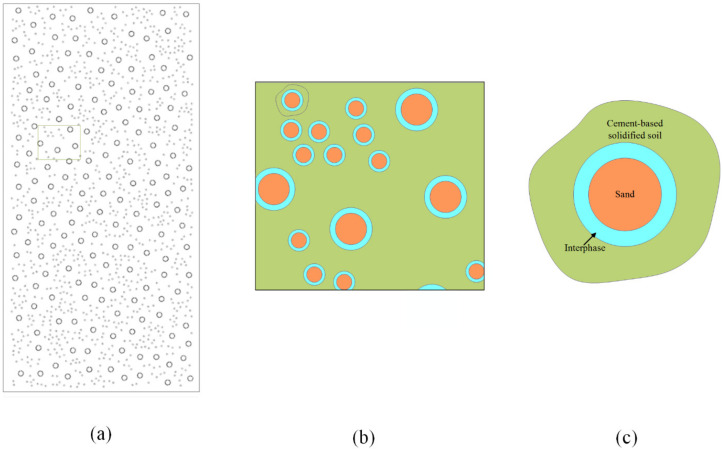
Global and local finite element models of CBSSM (**a**) Global Model of CBSSM (**b**) Local Model of CBSSM (**c**) Three-phase structure diagram.

This work actually generates a two-dimensional mesoscale representation of composites in Python by the Monte Carlo method [13], which refers to the programming language for scripting. The whole process is divided into two steps. The first step is to obtain a size table of randomly distributed aggregates following a certain grade curve. The second step is to put the aggregates one by one into a frame with a height of 10 cm and a width of 5 cm, while ensuring that they do not overlap with the previously placed particles and the boundaries of the frame. After finishing the code of the geometric model in python, transfer to the finite element calculation platform Abaqus to obtain the geometric model. Endow it material properties, boundary conditions, mesh, and compute it. In this work, the mesostructure of the CBSSM with 48% aggregate volume fraction was produced, as shown in Figure 7a. Next, four other numerical models following different gradation curves were created in the same way.

In Figure 7c, the outermost shell is on behalf of the paste cluster, where silt, clay and hydration products are considered as the phase. Their contributions to the mechanical properties of composite materials act as the CBSS. For sand phase, the diameter of the sand is roughly determined as 1.25 mm and 0.625 mm with reference to Table 1 and Figure 1, and the calculated sand numbers from the different gradation curves are 1156, 2286, 2853, 3301 and 3953, respectively. With these geometrical characteristics, the numerical structural models appeared. This method furnishes two main superiorities. Firstly, the numerical structural models can easily and rapidly be obtained. Secondly, setting a minimum inter-particle spacing ensures the quality of the model. Furthermore, take the ratio of interphase thickness to particle radius as 0.1 [34,35], which also delimits the distribution of the interphase.

**Figure 8:**
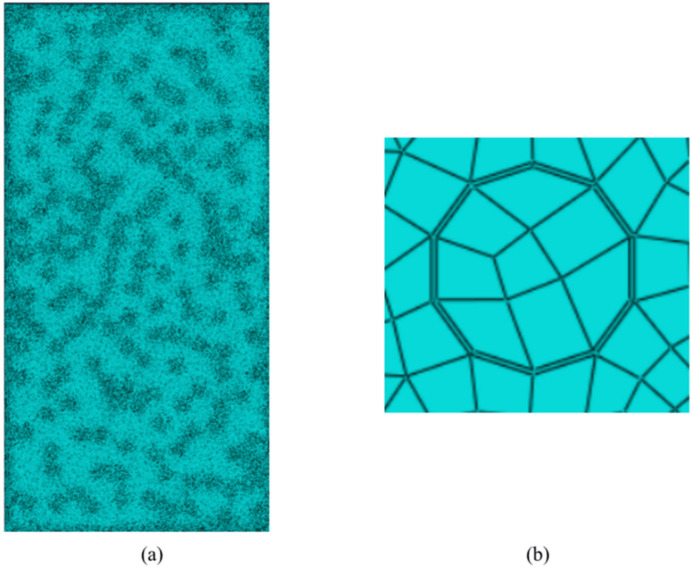
Global and local finite element meshes for CBSSM: (**a**) CBSSM (**b**) Interphase.

The mesh construction determines the analytical quality of the microstructure, which alters the accuracy of the numerical simulation. The internal length scale factor b can be considered as a regularization or a material length scaling factor. Refined mesh abides by (h ≤ ($\frac{1}{5}$~$\frac{1}{10}$)b) in the damage zone. However, thanks to the extremely small interphase thickness in this paper, the over-refinement of the mesh will greatly enlarge the calculation time and magnify the calculation cost. The advantages of using cohesive elements are highlighted. Although the middle surface of the cohesive element can withstand tensile and shear strains, it cannot generate any stress. Therefore, the cohesive element can only support the traction–separation failure criterion perpendicular to the upper and lower surfaces (traction–separation laws), which provides a solution without mesh refinement, which can effectively reduce the number of meshes and improve computational efficiency. The interphase behavior was described by a bilinear CM, and the element type of the interphase is four-node two-dimensional cohesive element (COH2D4). The global mesh size is 0.25, and the number of cloth types in the thickness direction of the interphase needs to be additionally set to 1. A sweeping method is employed to divide the quadrilateral meshes and the sweeping direction is along the thickness of the interphases. In order to improve the convergence, set the interphase viscosity coefficient to 0.001. The element type of the sand phase and the CBSS is CPS4R, which is a four-node bilinear plane stress quadrilateral element, and reduction integration and advanced algorithms are chosen. See the meshes of CBSSM and interphase in Figure 8a,b.

Figure 7a provides the two-dimensional numerical model of the CBSSM. To simulate the same boundary conditions as the test, the bottom of the model is fixed, and the top of the model bears a vertical compressed displacement. Suppose the sand is linear elastic and select its two material parameters namely elastic modulus and Poisson’s ratio according to sand type, which are given by the sand mechanical test. Then, pick the CDP model for the CBSS and reasonable selection of the following parameters was implemented according to the paper [32]. The two elastic parameters and the peak stress are directly defined through the mechanical test of the CBSS. Additionally, choose the two parameters $\sigma_{b0}/\sigma_{c0}$ and ξ of CBSS from the paper [32]. Moreover, we applied the sensitivity analysis to determine the other three parameters $K_{c}$, μ and ψ of the cement-based matrix. Within a reasonable range of parameters, adjust the three parameters in turn to fit the experimental curve. Generally, the dilation angle of cement-based brittle materials can be taken as 30°–39° [36], $K_{c}$ is used to define the shape of the yield surface and varies from 0.5 to 1 [37]. The viscosity parameter associated with the numerical convergence is defined as 0–0.01 [38,39]. According to the reliable value range, with the help of the sensitivity analysis, these three parameters have been successively corrected and listed in Table 2. 

## 5. Determination of Interphase Parameters 

In a 2D model, two individual fracture modes and their mixity, i.e., mode I (open mode), mode II (shear mode) and mixed mode, draw the fracture failure under general loading conditions. Additionally, each fracture mode of a certain material is accompanied by three parameters namely penalty stiffness, strength and fracture toughness. This paper only pays close attention to the elastic and damage stages of the interphase, so the four parameters garnered all the focus. Get the four interphase values by fitting the stress–strain curves from four unconfined compressive strength tests. Firstly, select the initial elastic constants ($E_{nn}$ and ν) by fitting the linear part of the stress–strain curves. The normal modulus $E_{nn}$ vary from 60% to 100% of the CBSS ($E_{m}$) [35], and the Poisson’s ratio fluctuates in the region of 0.35 [40]. Then, similarly identify the two damage parameters ($t_{n}^{0}$ and $t_{s}^{0}$) by onset point of variation in elastic properties in unloading path. Du and Jin [41] appraised that the interphase normal peak stress is usually 66%~88% of the tensile strength of CBSS. In addition, the interphase tangential peak stress can be addressed by the ratio ($t_{s}^{0}/t_{n}^{0}$) range [0.5, 5] [42]. Four sets of interphase parameters are listed in Table 3 by fitting the numerical and experimental results. All symbols and their meanings are listed in Table 4. 

The numerical stress–strain curves for different gradation curves were given in Figure 3, the errors of macro elastic modulus and unconfined compressive strength caused by the prediction are less than 20%, which implies the rationality of the interphase parameters. Although the apparent error of unconfined compressive strength (UCS) emerges in grade 3, the slight difference, due to experimental operation error, is acceptable. In addition, all arithmetic averages of interphase parameters are proven to be reliable in Figure 4. The specific interphase parameters are listed in Table 3.

The numerical result and test result of grade 5 is expressed in Figure 4, the errors of the macro elastic modulus ($E_{c}$) and unconfined compressive strength (UCS) are 0.07% and 7%. Obviously, the numerical result of UCS is lower than the experimental result. The reason explains diverse shapes and roughness of the sand are not taken into account in the numerical simulation [43], so that the action of friction is less considered [44].

## 6. Numerical Analysis and Discussion 

### 6.1. Average Interphase Thickness

Interphase geometry properties causes fluctuations in the macro mechanical behaviors of CBSSM. For the five numerical models for different particle gradation, the sand and interphase volume fractions, However, the number of interphases differs owing to different gradation curves, the number of interphases and sand particles is equal. According to the calculation, from grade 1 to grade 5, the number of sand particles gradually rises. Figure 5 revealed the downward trend of the macro elastic stiffness along with the quantity of sand particles gradually expands in the light of the gradation information in Figure 1. Similarly, an obvious downward trend of unconfined compressive strength emerges in Figure 6. These observations can be explained by the growing number of interphases and the decrease of the average interphase thickness. It should be noted that in the linear framework, this above explanation has been approved on basis of the relationship between the interphase thickness and the spring interphase model [27]. 

### 6.2. Interphase Modulus

The two-dimensional composite material cracking includes generally open and slip types; thus, the normal elastic behavior and the tangential elastic behavior of the interphase cannot be underestimated. Referencing the paper [36], the interphase normal elastic modulus is lower than that of the cement paste, so select the ratio $E_{nn}/E_{m}$ with values of 0.1, 0.5 and 1.0 for the numerical simulation.

**Figure 9:**
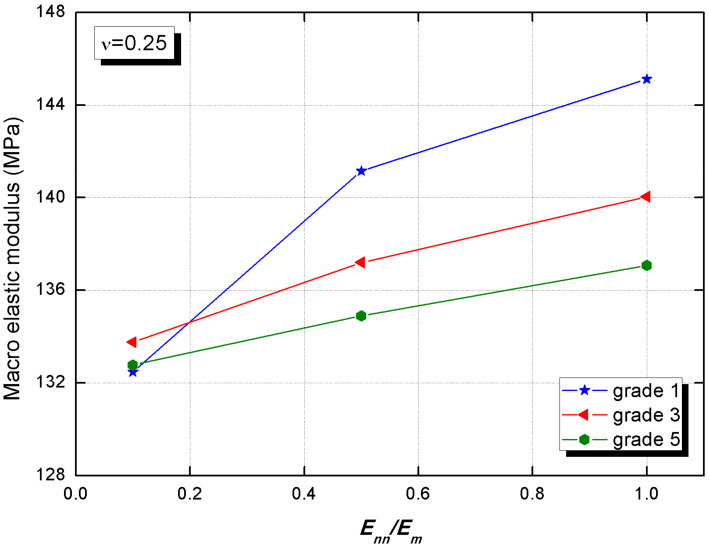
The macro elastic modulus of CBSSM versus the normal elastic modulus of the interphase.

Figure 9 clears that the upward floating of interphase normal elastic modulus causes a distinct nonlinear macro behavior, which can be certified by the theoretical analysis from the Hashin model and the Ramesh model [45]. It is because the improvement in interphase elastic modulus causes an expansion in interphase stiffness, which possibly enhances the ability to transmit force and resist deformation between the interphase and adjacent phases. Some other experiments observed that adding fine particles can be used to change the hydration product density which is beneficial to the interphase properties [46]. Furthermore, from Figure 9 we notice that the macro elastic stiffness rises by 9.5% and 5.8%, caused by the normal elastic modulus and the average thickness of interphases, respectively. This demonstrates that the effect of the interphase normal elastic modulus is more significant.

**Figure 10:**
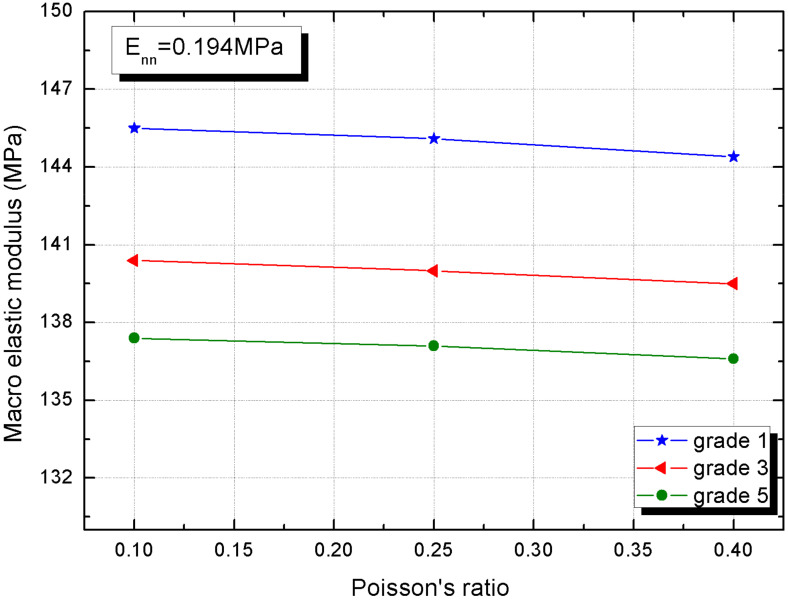
The macro elastic modulus of CBSSM versus the Poisson’s ratio of the interphase.

The effect of interphase tangential elastic modulus on the macro behavior can be described by Poisson’s ratio referring to Equation (2) with the constant normal elastic modulus. Figure 10 hints the slightly reduced trend of macro elastic modulus, which confirms the interphase tangential elastic modulus contributes little to the macro elastic modulus. The open type is without a doubt the main deformation mode of interphase. In addition, the macro elastic modulus increment (0.8%) indicates that the role of interphase tangential elastic or Poisson’s ratio is minimal. Wang et al. [39] also offered similar conclusions with different materials.

### 6.3. Interphase Peak Stress

**Figure 11:**
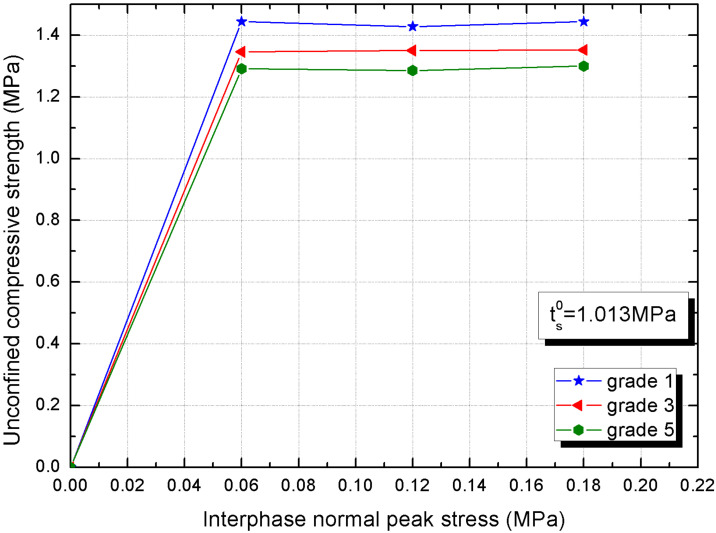
Unconfined compressive strength (UCS) versus interphase normal peak stress.

**Figure 12:**
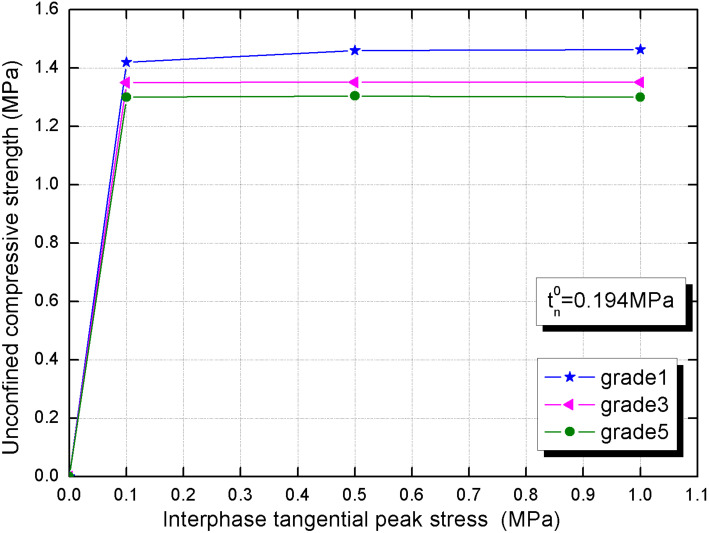
Unconfined compressive strength (UCS) versus interphase tangential peak stress.

The link between two interphase damage property parameters, namely interphase normal and tangential peak stresses, and macro compressive strength of CBSSM are researched. The unconfined compressive strength firstly goes up significantly with the rise of the interphase normal peak stress and then almost remains stable in Figure 11, the critical value is 0.0194 MPa, which is about 2.5% of the cement paste tensile strength. This is because the interphase is essentially the accumulation of hydration products, and when the hydration product transmits force and deformation excellently, the interphase reaches a critical peak stress and the volatility tends to stabilize [46]. In fact, from Figure 11 and Figure 12, we observe that the macro compressive strength is the largest in grade 1 and the smallest in grade 5. This is because of the variation in the average thickness of the interphase, grade 1 has fewer interphases than grade 5. Thus, the fact that there is greater average interphase thickness makes more sense for CBSSM.

In Figure 12, with the ascent of the tangential peak stress, we can also observe that the unconfined compressive strength (UCS) heightens first and then stabilizes, whose critical peak stress value is 1.013 MPa. We can see that the ability of the interphase to resist slippage is basically formed, that is, the compactness of the hydration product grains in the shear direction ensures the transmission of force and deformation. Similarly, when the tangential shear stress is sufficient, the greater average interphase thickness contributes to CBSSM.

## 7. Conclusions

At the microscopic level, we can look at the CBSSM as a three-phase composite material including sand, CBSS and interphase with thin thickness. The weakest interphase acts very clearly on the strength and stiffness of the CBSSM. Firstly, this present paper probes the interphase behavior in CBSSM with tests. Then, based on the geometric characteristics of the experimental specimen, the numerical models were produced. By fitting the experimental and numerical results, the interphase parameters are determined and verified. Finally, a sensitivity analysis of interphase parameters is conducted. Numerical prediction results can draw the following conclusions:

1. By comparing with experimental results, the errors of the macro elastic modulus and peak compressive strength are limited to be about 0.07% and 7%, indicating that the numerical model and the selected interphase parameters can elaborate the interphase behavior well.
2. When the volume fraction of the interphase is constant, the macro elastic modulus and unconfined compressive strength (UCS) decreases with average interphase thickness declining.
3. The ascension of the macro elastic modulus, caused by interphase normal and tangential elastic modulus, is 9.5% and 0.8%, respectively,
4. As the normal and tangential peak stress of the interphase rise, the CBSSM demonstrates higher compressive strength, while they exceed these critical values, the unconfined compressive strength remains almost constant.
5. For the macro elastic modulus of CBSSM, the normal elastic modulus and the average interphase thickness play a significant role. For its unconfined compress strength, the normal and tangential peak stress effects are apparent at the initial stage, while the effect of average interphase thickness is more notable at the constant stage.

The above results illustrate that in practical engineering, the interphase thickness, elastic modulus and strength of composite materials are extraordinary factors. In order to weaken the adverse effects of interphase, it may be effective to agitate the components of the mixture well and carry out standard curing. This will increase the durability and service life of the composite material to a certain extent, delaying the generation of damage and even cracks.

## Figures and Tables

**Table 1:** Gradation information of sand for numerical simulation.

	Particle Size (mm)	2	0.5	0.075
Gradation				
1		100.0	73.0	64.0
2		100.0	83.8	64.0
3		100.0	89.2	64.0
4		100.0	93.5	64.0
5		100.0	99.7	64.0

**Table 2:** Material parameters of cement-based solidified clay matrix and sand.

Specimen	Cement-Based Solidified Clay Matrix								Sand	
	$\sigma_{b0}/\sigma_{c0}$	$K_{c}$	ϕ	ξ	μ	$\nu_{m}$	$E_{m}\;(\mathrm{MPa})$	$\sigma_{c0}\;(\mathrm{MPa})$	$\nu_{a}$	$E_{a}\;(\mathrm{MP})$
1–5	1.16	0.667	39°	0.1	0.01	0.30	145.3	1.22	0.2	500

Note: $\sigma_{b0}/\sigma_{c0}$ = ratio of biaxial compressive to uniaxial compressive yield stress; $K_{c}$ = coefficient ascertain; ϕ = dilation; ξ = potential flow eccentricity; μ = relaxation time; $\nu_{m}$ = cement-based solidified clay Poisson’s ratio; $\nu_{a}$ = sand Poisson’s ratio; $E_{m}$ = cement-based solidified clay elastic modulus; $E_{a}$ = sand elastic modulus.

**Table 3:** Interphase parameters.

Gradation	$E_{nn}\;(\mathrm{MPa})$	ν	$t_{n}^{0}\;(\mathrm{MPa})$	$t_{s}^{0}\;(\mathrm{MPa})$
1	130	0.24	0.190	0.950
2	145	0.27	0.200	1.000
3	145	0.26	0.220	1.100
4	130	0.23	0.200	1.000
Average	137.5	0.25	0.194	1.013

Note: $E_{nn}$ = normal elastic modulus; ν = Poisson’s ratio; $t_{n}^{0}$ = normal peak stress; $t_{s}^{0}$ = tangential peak stress.

**Table 4:** Symbols and meanings in the paper.

Symbols	Meanings
$K_{c}$	coefficient ascertain
ϕ	dilation
ξ	potential flow eccentricity
μ	relaxation time
$\nu_{m}$	cement-based solidified clay Poisson’s ratio
$\nu_{a}$	sand Poisson’s ratio
$E_{m}$	cement-based solidified clay elastic modulus;
$E_{a}$	sand elastic modulus
$E_{nn}$	normal elastic modulus
ν	Poisson’s ratio
$t_{n}^{0}$	normal peak stress
$t_{s}^{0}$	tangential peak stress
$\sigma_{b0}/\sigma_{c0}$	ratio of biaxial compressive to uniaxial compressive yield stress

## Data Availability

The data presented in this study are available on request from the corresponding author.
